# Virtuelle Stabsarbeit in Zeiten der Pandemie – Entwicklung digitaler Übungsformate in der zivilen Gefahrenabwehr während der Coronapandemie: Erfahrungen am Beispiel des Projekts ARMIHN

**DOI:** 10.1007/s10049-023-01164-7

**Published:** 2023-06-15

**Authors:** Julian Heuser, Boris Tolg, Karsten Loer, Angelina Klein, Nadine Sprössel, Jonas Klein, Lyubomir Haralambiev, Marcus Oldenburg, Kristina Carolin Militzer, Lukas Belz, Thomas von Münster, Volker Harth, Lena Ehlers, Jens de Boer, Scarlett Kleine-Kampmann, Matthias Boldt, Martin Dirksen-Fischer, Markus Wiedemann, Axel Ekkernkamp, Mustafa Sinan Bakir

**Affiliations:** 1 Zentrum für Orthopädie, Unfallchirurgie und Rehabilitative Medizin, Universitätsmedizin Greifswald (UMG), Ferdinand-Sauerbruch-Straße, 17475 Greifswald, Deutschland; 2 Abteilung Medizintechnik, Hochschule für Angewandte Wissenschaften Hamburg (HAW Hamburg), Hamburg, Deutschland; 3 Zentralinstitut für Arbeitsmedizin und Maritime Medizin (ZfAM), Universitätsklinikum Hamburg-Eppendorf (UKE), Hamburg, Deutschland; 4 Hamburg Port Health Center (HPHC), Institut für Hygiene und Umwelt, Hamburg, Deutschland; 5 Klinik für Unfallchirurgie und Orthopädie, BG Unfallkrankenhaus Berlin gGmbH, Berlin, Deutschland

**Keywords:** MANV, MANE, Simulation, Digitale Plattform, Endnutzerakzeptanz, MCI, MCI-ID, Simulation, Digital platform, End-user acceptance

## Abstract

**Hintergrund:**

Im Bereich der Gefahrenabwehr von Großschadensereignissen, wie einem Massenanfall von Verletzten (MANV) oder von Erkrankten (MANE), sind regelmäßige Übungen essenziell, um im Ereignisfall erfahrene Einsatzkräfte zur Verfügung zu haben. Pandemiebedingt mussten Übungen häufig abgesagt werden oder waren nur in kleinen Personengruppen möglich. Die Abbildung einer Großschadenslage mit Darstellern war häufig nicht möglich, sodass den Übenden keine realitätsnahen Szenarien eines MANV oder MANE angeboten werden konnten. Im Rahmen zweier Forschungsprojekte wurde zur Vermeidung eines Infektionsrisikos von Übungsteilnehmenden eine digitale Plattform zur Durchführung von Übungen genutzt, um den Personaleinsatz vor Ort auf ein Minimum reduzieren zu können. Ziel dieser Arbeit war die Evaluation der digitalen Lösungsansätze in Bezug auf ihre Endnutzerakzeptanz.

**Methoden:**

Im Rahmen des Projekts „Adaptives Resilienz Management im Hafen“ (ARMIHN) wurde eine digitale Übungsplattform angewendet und mithilfe von Teilnehmendenbefragungen nach den Schwerpunkten „Umsetzung“, „Alternativmöglichkeiten“, „Lerneffekt“ und „Nutzbarkeit“ evaluiert. Die Teilnehmenden nutzten die digitale Plattform zum Informationsaustausch sowie zur Kommunikation. Hierfür wurden verschiedene Kollaborationstools in die Plattform eingebettet, welche einen simultanen Austausch von Informationen in Echtzeit ermöglichten. Eine stetige Videokommunikation zu eigenen und auswärtigen Kräften wurde ebenfalls etabliert.

**Ergebnis:**

Das Potenzial der digitalen Plattform als Alternative zu Übungen in Präsenz wurde durch die beteiligten Endanwender im ARMIHN-Projekt mit 90 %iger Zustimmung bestätigt. Auch die Steigerung der subjektiven Fähigkeiten und des Kenntnisgewinns bei einem MANE wurde überwiegend zustimmend bewertet (bis zu 70 %). Teilnehmende, die die Umsetzung des Online-Formats als gut gelungen bewerteten, gaben signifikant häufiger an, dass sich subjektiv ihre Fähigkeiten zum Umgang mit einem MANE verbessert hätten (*p* = 0,016). Dahingegen wurde eine virtuelle Stabsarbeit in realen Krisensituationen von ungefähr der Hälfte der Befragten kritisch gesehen.

**Diskussion:**

Die Evaluationsergebnisse weisen insgesamt auf die hohe Endnutzerakzeptanz des entwickelten Konzepts hin. Auch wenn angestrebt ist, das System über einen längeren Zeitraum mit größerer Anzahl von Teilnehmenden weiter zu evaluieren, so bestätigen die bereits durchgeführten Untersuchungen die positiven Erfahrungen in den jeweiligen Projekten.

**Zusatzmaterial online:**

Die Online-Version dieses Beitrags (10.1007/s10049-023-01164-7) enthält den zugrunde liegenden Fragebogen.

## Abstract (en)

**Background:**

In the event of emergency response to large-scale incidents, such as a mass casualty incident (MCI) or a mass casualty incident–infectious disease (MCI-ID), regular training is essential in order to have experienced emergency personnel available in the event of an incident. Due to the pandemic drills often had to be cancelled or were only possible with small groups of people. It was often not possible to simulate a large-scale emergency with actors, so that the trainees could not be offered realistic scenarios of a mass casualty or disease incident. As part of two research projects, a digital platform for conducting training was used to avoid the risk of infection between participants during the exercises, so that on-site personnel deployment could be reduced to a minimum. The goal of this work was to evaluate end-user acceptance of the digital solution approaches.

**Methods:**

Within the framework of the project “Adaptive Resilience Management in Ports” (ARMIHN), a digital exercise platform was applied and evaluated with the help of participant surveys according to the focal points “implementation”, “alternative possibilities”, “learning effect” and “usability”. The participants used the digital platform to exchange information and to communicate. For this purpose, various collaboration tools were embedded in the platform, which enabled simultaneous exchange of information in real time. Constant video communication with in-house and external authorities/teams was also established.

**Results:**

The potential of the digital platform as an alternative to on-site exercises was confirmed by the participating end users in the ARMIHN project with 90% agreement. The increase in subjective skills and knowledge gained during a MCI-ID was also predominantly rated approvingly (up to 70%). Participants who rated the implementation of the online format as well performed were significantly more likely to state that subjectively their ability to handle a MCI-ID had improved (*p* = 0.016). In contrast, virtual staff teamwork in real crisis situations was viewed critically by about half of respondents.

**Conclusion:**

Overall, the evaluation results point to the high end-user acceptance of the developed concept. Even though the aim is to evaluate the system over a longer period with a larger number of participants, the studies already conducted confirm the positive experiences in the respective projects.

## Hintergrund

Die gesellschaftlichen und wirtschaftlichen Folgen der COVID-19-Pandemie sind weltweit allgegenwärtig spürbar gewesen. Die Pandemie zeigte ihre Relevanz auch besonders deutlich in der maritimen Medizin. Berichte über COVID-19-Ausbrüche an Bord von Schiffen, wie beispielsweise auf dem Passagierschiff „Diamond Princess“ [1, 2], zeigen das hohe Übertragungsrisiko von Atemwegserkrankungen an Bord [3]. In diesem Fall war die Reproduktionszahl (R_0_ = 14,8) initial vierfach höher als in Wuhan [2]. Die Umsetzung von Isolations- und Quarantänemaßnahmen an Bord ist aufgrund der räumlichen und technischen Gegebenheiten (Enge, raumlufttechnische Anlagen) oft problematisch. Zur Reduktion des Infektionsrisikos, zur Behandlung von komplizierten Verläufen und mit Blick auf die psychosoziale Betreuung, ist die zeitnahe Evakuierung aller Personen an Bord erstrebenswert. Die Effektivität der bisherigen Quarantänemaßnahmen im maritimen Setting war diesbezüglich in der Vergangenheit durchaus umstritten [2, 4, 5].

Simulationsübungen mit mehreren Personen und Gruppen an einem Ort waren pandemiebedingt (Abstands- und Hygieneregeln) nahezu unrealisierbar. In der Gefahrenabwehr sind diese jedoch als adäquate Vorbereitung für mögliche Schadensfälle regelmäßig zwingend durchzuführen [6, 7].

Übungen zu einem Massenanfall von Verletzten (MANV) oder einem Massenanfall von Erkrankten (MANE) waren organisatorisch und personell stets aufwendig [8–10]. Deren Umsetzbarkeit wurde pandemiebedingt noch schwieriger und allenfalls in Kleingruppen möglich. Dies konterkariert jedoch die Simulation eines MANE, deren Problemstellung das unmittelbare Missverhältnis zwischen Einsatzaufkommen und verfügbaren Kräften ist.

Ziel des Projekts ARMIHN (Adaptives Resilienz Management im Hafen), gefördert durch das Bundesministerium für Bildung und Forschung (BMBF; Förderkennzeichen 13N14923 – 13N14925), war die Verbesserung der Vor-Ort-Resilienz und Handlungsfähigkeit beim MANE als infektiologische Notfallsituation im Hafen. Hierfür wurden ein Konzept zur Bewältigung sowie ein adaptives Trainingskonzept entwickelt. Beides wurde im Rahmen von drei Stabsübungen und einer Vollübung im Hamburger Hafen erprobt, welche ursprünglich allesamt in Präsenz konzipiert waren. Die Fachstäbe sollten gemeinsam in einem Raum zusammenkommen und hätten mithilfe von Planspielen und Simulationen die Gefahrenabwehrmechanismen gemeinsam geübt. Die bisherigen Übungsverfahren für Patientensimulationen waren hierbei bisher entweder papierbasiert oder mussten aufwendig mithilfe von Laienschauspielern organisiert werden. Beides ist vor allem in der Situation einer anhaltenden Pandemielage mit Schwierigkeiten verbunden: Der papierbasierte Ansatz kann den dynamischen Herausforderungen kaum gerecht werden, der Umgang mit vielen Simulationspatienten stellt, neben dem großen organisatorischen Aufwand und hohen Kosten, ein weiteres Infektionsrisiko dar.

### Fragestellung

Um die regelmäßigen, notwendigen Übungen für die essenziellen Gefahrenabwehrbehörden umsetzen zu können, soll die Hypothese untersucht werden, dass die eingesetzten digitalen und virtuellen Einsatzformate in der Stabsarbeit von den Teilnehmenden als adäquate Antworten auf die pandemischen Rahmenbedingungen angesehen werden. Dafür wurde analysiert, inwieweit diese von den Nutzern als gute Alternative für die neuen Herausforderungen akzeptiert und als hilfreich bewertet wurden.

## Studiendesign und Untersuchungsmethoden

**Figure 1:**
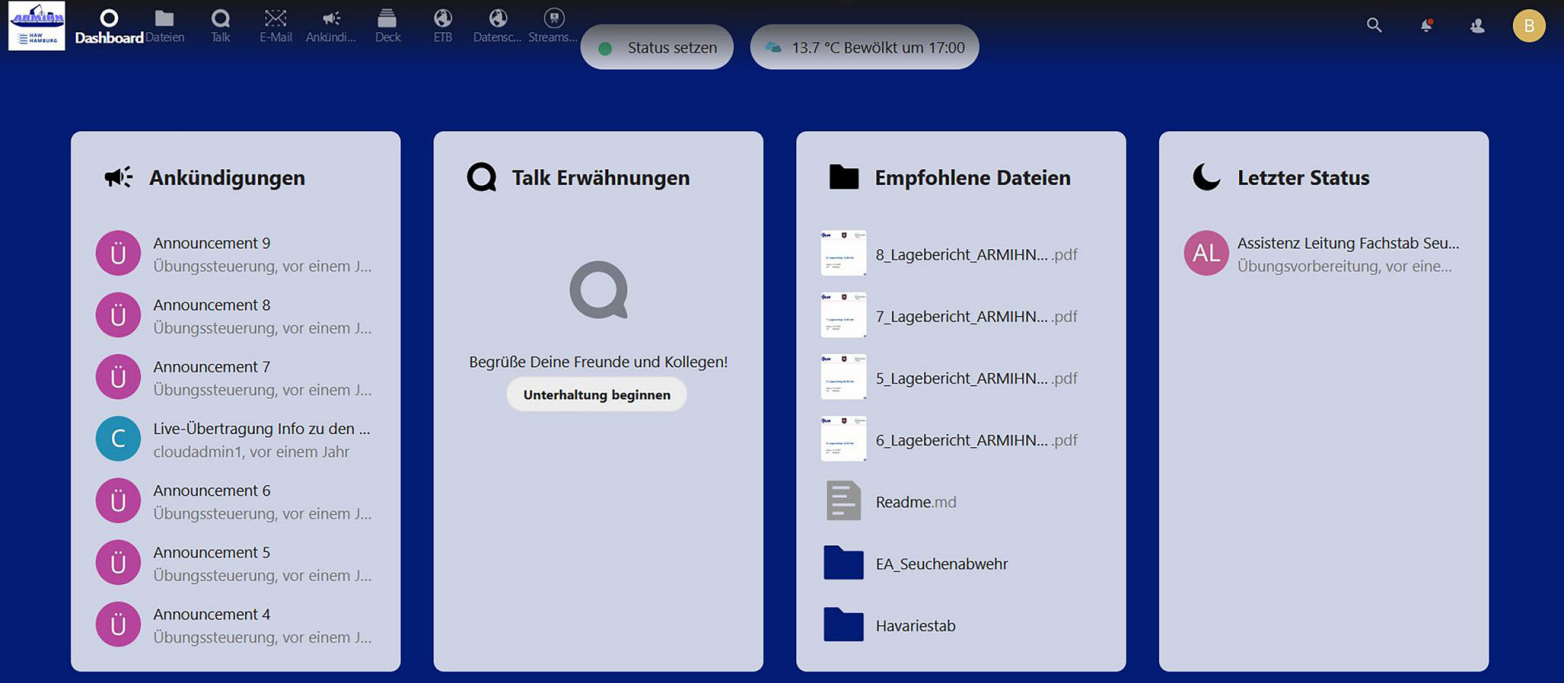

Die digitalen Übungen im Projekt ARMIHN wurden über eine von der HAW Hamburg für die Forschung und Lehre weiterentwickelte Nextcloud-Plattform zur Unterstützung digitaler Stabsrahmenübungen durchgeführt (Abb. 1; [11]).

Dieses digitale Format wurde für das Projekt mit weiteren Funktionalitäten erweitert. Das daraus resultierende Zusammenspiel zwischen digitaler Plattform und eingebetteten Cloud-Anwendungen wurde als zentrales Element der technischen Umsetzung entwickelt, um eine effiziente Stabsarbeit mit geeigneten und intuitiv bedienbaren digitalen Hilfsmitteln zu unterstützen. So wurden Funktionen aus bestehender Software kombiniert oder gänzlich neu geschaffen. Dies betraf die Kommunikation der Übungsteilnehmenden, deren Arbeit mit Tabellen und Textdokumenten, die Arbeitsplanung und Maßnahmenverfolgung und die Schaffung eines gemeinsamen Lagebilds (Tab. 1). Nicht alle der wesentlichen Elemente eines virtuellen Stabraums (wie beispielsweise Whiteboard, Videokonferenz und dynamische Lagedarstellung) wurden als digitale Werkzeuge auch verwendet, während sie jedoch vollumfänglich in die digitale Plattform integriert sind. Welche Elemente im Rahmen der ARMIHN-Übungen aktiv genutzt wurden, ist in Tab. 1 dargestellt.

**Table 1:** 

Begebenheiten in „physischem Stabsraum“	Digitale Abbildung dieser Begebenheiten	Derzeit eingebundene Werkzeuge	dModTTX			ARMIHN		
			Teilnehmende	Übungssteuerung	Beobachtung/Zertifizierung	Teilnehmende	Übungssteuerung	Beobachtung/Zertifizierung
*Kommunikation*								
Übungssteuerung Kommunikation innerhalb Stabsbereichen Kommunikation Stab↔Außenwelt	Textnachrichten	Talk^a^	x	–	–	x	x	–
	Plattforminternes E‑Mail-System (autom. Kopien jeder E‑Mail an Protokoll-Account)	Mail^a^	x	–	x	x	–	x
	Einzel‑/Gruppengespräche	Talk^a^, Wonder.me	x	x	x^b^	–	–	–
	Adressbuch	Kontakte^a^	x	x	–	–	–	–
	Zugang zu Videoconferencing	Jitsi-Server, Webex, Zoom, MS Teams	Jitsi, Zoom	–	x^b^	Webex, Zoom	–	–
*Arbeit mit Dokumenten*								
Modellierung verschiedener Kommunikationswege innerhalb des Stabs und zwischen Stab und Außenwelt (Meldungen/Nachrichtenvordruck, Fax, Pressemitteilungen, Briefe, Social Media …) Verfassen von Antworten Arbeitsorganisation innerhalb der Stabsbereiche	Dateiablage	Nextcloud	x	x	x	x	x	x
	Kollaborative Office-Anwendungen	ONLYOFFICE	x	x	x	x	x	x
	Erstellung von Grafiken, Organigrammen, Ablaufplänen	Draw.io	–	–	–	–	–	–
	Gruppenrichtlinien	Nextcloud	x	x	x	x	x	x
*Gemeinsames Lagebild*								
Priorisierung übergeordneter Ziele Lagekarte Wetter	Whiteboard	Whiteboard	–	–	–	–	–	–
	Digitale Lagekarte	Mobile-Lagekarte.de	Statische Präsentation	–	x (lesend)	Statische Präsentation	–	–
	Einsatztagebuch	Tabellendokument	–	–	–	x	x	x
*Arbeitsplanung*								
Priorisierung übergeordneter Ziele Aktuelle Einsätze „Warteliste“ zu bearbeitender Einsätze Kommunikationsplan	Whiteboard	Whiteboard-Server	–	–	–	–	–	–
	Kanban-Board	Deck^a^	–	–	–	–	–	–
	Kalender	Kalender^a^	–	–	–	–	–	–
	Kommunikationsplan	Draw.io	–	–	–	–	–	–
*Übungssteuerung*								
Besprechungen des Übungsablaufs und -fortschritts Situative Einspielungen vorbereiteter Übungselemente (Nachrichten, Informationen, Schriftstücke …) Situative Einspielung von Live-Übungselementen Kommunikation im Spiegelstab Ortsabhängiger Zugriff auf taktische Zeit „Bildregie“ für externe Beobachtende	Durchsagen (systemweit)	Announcements^a^	–	x	x	–	x	–
	Durchsagen (ausgewählte Nutzergruppen)	Talk^a^, Announcements^a^	–	x	–	–	x	–
	Live-Einspielungen	Announcements^a^, privater YouTube-Kanal	–	x	–	–	x	–
	GreenScreen	Eigenentwicklung	–	x	–	–	x	–
	Taktische Zeit	Eigenentwicklung	x	x	x	x	x	x
*Übungsauswertung*								
Regelmäßige fragebogenbasierte Messung der Selbsteinschätzung Auswertung und Besprechung am Ende jedes Übungstags bzw. bedarfsweise während der Übung Verfolgungsmöglichkeit für externe Übungsbeobachtende	„Freeze“-Funktion	Eigenentwicklung	–	–	–	x	x	–
	Fragebögen (NASA TLX, …)	MS Forms	–	–	–	x	x	–
	Einblick in sämtliche schriftliche Kommunikation	–	–	x	x	–	x	x
	Übertragung der Videokonferenzen	Privater YouTube-Kanal	–	x	x^b^	–	x	x

^a^Nextcloud-Plugin

^b^Spiegelung der Videokonferenzen in private YouTube-Kanäle für externe Beobachtende

Für die Übungssteuerung und -bewertung durch externe Nutzer bzw. spätere Auswertung wurden zudem Möglichkeiten zur Mitverfolgung der schriftlichen Kommunikation und Videokonferenzen geschaffen. In der Entwicklung des digitalen Formats wurde darauf Wert gelegt, bevorzugt auf frei verfügbare Open-Source-Software zurückzugreifen, um eine spätere Verbreitung der Software durch einen geringen IT-Aufwand sowie relativ geringe Kosten zu ermöglichen.

Regelmäßige Online-Befragungen der Teilnehmenden während der Übung ermöglichten die Bewertung ihrer Fortschritte. Dies erfolgte durch 5‑minütige Befragungen, während der Zugriff auf die Plattform zeitgesteuert verhindert wurde. Während der Pausen wurde anstelle der Plattform ein Link auf die aktuelle Befragung angezeigt. Nach den Pausen wurde die taktische Zeit bei Bedarf durch die Software automatisch angepasst.

ARMIHN war eine prospektive Studie vom 01.03.2019 bis 31.12.2021. Stufenweise erfolgten Literaturrecherche, Strukturanalysen mit Fachexperten, Entwürfe von Kommunikationsleitfäden, Einsatzszenarien und -strategien, Vorbereitung und Durchführung von MANE-Übungen und deren Evaluation. Die Übungsformate wurden mithilfe der digitalen Plattform unter Pandemiebedingungen weiterentwickelt. Neben den beschriebenen virtuellen Stabräumen wurden digitale Evaluationen und Patientenkarten zur Triage erstellt (weiterführende Informationen unter www.armihn.de).

Die drei zwischen Juni und August 2021 durchgeführten Stabsübungen fokussierten sich auf intra- und interorganisationelle Kommunikation, Krisenmanagement, Raumordnung und Triage (Seuchen- und Schadensabwehr). Eine Hybridübung zur MANE-Simulation im Hamburger Hafen erfolgte vor Ort im Oktober 2021. Für die vier Übungen wurden verschiedene Einsatzszenarien erstellt, welche die Komplexität eines MANE abbildeten. Ein Einsatzszenario stellte das maximale Missverhältnis zwischen Patientenaufkommen und Einsatzkräften anhand einer gastrointestinalen Erkrankung an Bord eines Kreuzfahrtschiffs dar. Ein weiteres, hochdynamisches Szenario war ein MANE einer hochkontagiösen Atemwegsinfektion unter ähnlichen Bordbedingungen.

Die Teilnehmenden und die Evaluator*innen der Übung erhielten nach Abschluss der letzten Übung einen (digitalen) Fragebogen. Der Fragebogen umfasste vier Schwerpunkte:

- Evaluation der Umsetzung (Frage 1),
- Alternativmöglichkeit zur Präsenz (Frage 2 + 3),
- (Subjektiver) Lerneffekt (Frage 4 + 5),
- Nutzbarkeit der Plattform im Realfall (Frage 6 + 7).

Die Beantwortung erfolgte auf einer fünfgliedrigen Likert-Skala von „stimme voll zu“ bis „stimme überhaupt nicht zu“ (0, 1 = ablehnend; 3, 4 = zustimmend). Die beschriebenen Fragebögen sind im Supplement dieser Arbeit zu finden. Die Datenauswertung erfolgte mittels SPSS (IBM SPSS Statistics for Windows, Version 22.0, IBM Corp., Armonk, NY, USA). Korrelationen wurden mittels Pearson-Korrelationskoeffizienten (r) und Kendalls Tau (τ) analysiert, für vereinzelte Fragestellungen wurde der Chi-Quadrat-Test angewandt. Auf eine weitergehende, detaillierte statistische Analyse der Signifikanz wurde aufgrund der geringen Fallzahl verzichtet.

Die Studie ist im Deutschen Register Klinischer Studien (DRKS00022327) registriert und von der lokalen Ethikkommission der UMG (BB 051/19) genehmigt.

## Ergebnisse

**Figure 2:**
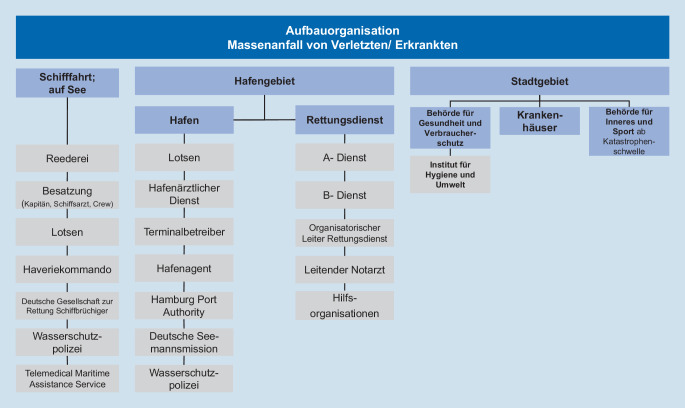

Vollständig beantwortet wurden *n* = 20 Evaluationen. Die Evaluator*innen und aktiven Teilnehmenden bewerteten insbesondere die digitalen Formate als neu, innovativ und funktional. Diese kamen aus unterschiedlichen Fachbereichen der beim MANE im Hafen beteiligten Organisationseinheiten: Schifffahrt, Hafen- und Stadtgebiet (Abb. 2; [12]).

**Figure 3:**
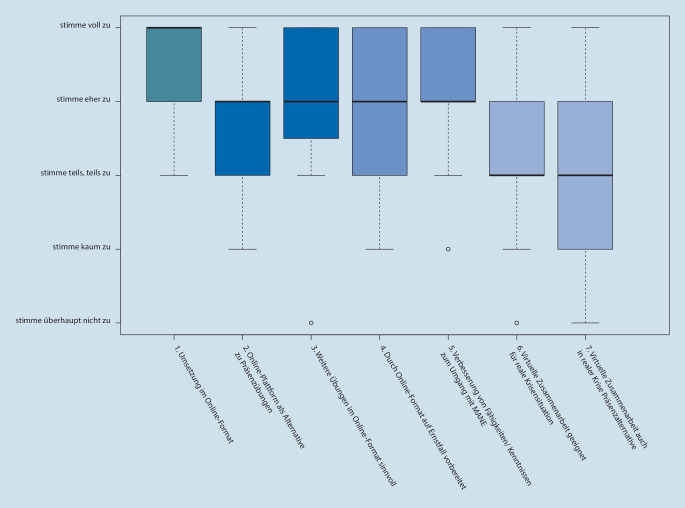

Das digitale Konzept wurde von 90 % als positiv evaluiert (Abb. 3, Frage 1). Die Antworten auf Frage 2 und 3, zur Online-Plattform als geeignete Alternative und als gewünschtes zukünftiges Format, zeigten eine signifikante Korrelation (*p* < 0,001, r = 0,777, τ = 0,711) in der Zustimmung (60 % bzw. 75 %). Der subjektive Lerneffekt (Frage 4 und 5) wurde ebenfalls positiv bewertet. Diese positive Einschätzung zeigte ebenfalls eine signifikante Korrelation zwischen den Fragen, wenn auch schwächer ausgeprägt (*p* = 0,001, r = 0,695, τ = 0,571). Fast zwei Drittel der Befragten sahen die Online-Plattform als gute Vorbereitung auf den Ernstfall und 70 % bewerteten ihre Fähigkeiten und Kenntnisse im Umgang mit einem MANE als verbessert. Letzteres wurde signifikant häufiger angegeben von Teilnehmenden, die die Umsetzung des Online-Formats als gut gelungen bewerteten (*p* = 0,016).

Die Fragen zur Nutzbarkeit der Plattform im Realfall (Frage 6 und 7) wurden weniger zustimmend bewertet und korrelierten signifikant miteinander (*p* < 0,001, r = 0,883, τ = 0,852). Zur virtuellen Zusammenarbeit als geeignete Alternative zur Präsenz in realen Krisensituationen (Frage 7) stimmten weniger als die Hälfte der Befragten zu, und ca. 30 % lehnten diese ab.

## Diskussion

Ein MANE ist charakterisiert durch ein Missverhältnis zwischen der Anzahl an Erkrankten und der Anzahl der verfügbaren Einsatzkräfte. Dies führt zum Aussetzen der individualmedizinischen Prioritäten und verlangt nach effizienter und stringenter Zusammenarbeit einzelner Stakeholder [13]. Elementare Voraussetzungen dafür sind notwendige Arbeitsroutinen und Kenntnis über die jeweiligen Organisationen [14], welche nur durch stetiges Üben etabliert werden können [8]. ARMIHN zeigte, dass auch in Pandemiezeiten mit notwendiger Kontaktreduzierung essenzielle Übungen mithilfe digitaler Formate umsetzbar sind. Die positiven Evaluationen zeigen das grundsätzliche Potenzial der genutzten digitalen Infrastruktur auch über die Pandemiebedingungen hinaus.

Oft besteht bei der Simulation von Großschadensereignissen eine Übungskünstlichkeit [6, 15, 16], die Einflüsse bei der Beobachtung oder Realitätsverzerrungen wie Zeitsprünge bei diesem Übungsformat nach sich zieht. Auf der anderen Seite sind Präsenzübungen mit enormem organisatorischem Vorlauf verbunden und in der Pandemie, mindestens in großem Ausmaß, nicht möglich gewesen. Der Vorteil des Formats liegt in der Aufrechterhaltung des gemeinsamen Übens, jedoch mit der Einschränkung fehlender sozialer und nonverbaler Kommunikation [17]. Die positive Selbsteinschätzung der Übungsteilnehmenden bezüglich des Lerneffekts gibt eine optimistische Bewertung ab. Allerdings wurden die Lerneffekte in den einzelnen Übungen nicht explizit überprüft und die Verfestigung des Wissenstransfers bedarf regelmäßiger Wiederholungen.

Ein wichtiger Einflussfaktor bei der Interpretation der Ergebnisse ist, dass bei den Übungen eine große Heterogenität der verschiedensten Fachbereiche der Teilnehmenden vorherrschte. Dies ist der Tatsache geschuldet, dass in Punkten der kritischen Infrastruktur stets eine Vielzahl an Stakeholdern und Akteuren zusammenkommen muss [18–20]. Für das erfolgreiche Gelingen von Testungen, Überprüfungen und der Durchführung von Übungen ist die Involvierung möglichst aller Player daher wichtig, da nur so realistische Bedingungen geschaffen werden können und Übungskünstlichkeiten möglichst vermieden werden. Durch die Partizipation von Organisationen, welche nicht primär in erster Reihe in das Management eines MANE eingebunden sind und durch die nachgeordnete Stellung im Einsatzfall noch weniger Erfahrung diesbezüglich haben, ist es möglich, dass eine adäquate Beantwortung der spezifischen Fragen daher nicht in jedem Fall durchführbar war. Insofern könnte das genutzte Format nicht für alle Personen ausreichend zur Simulation geeignet gewesen sein bzw. der Wissenstransfer nicht ausreichend gewährleistet worden sein. Obgleich der Großteil den subjektiven Lerneffekt bestätigte, wären hier weiterführende Untersuchungen, ggf. separiert nach entsprechender Organisationsgruppe, zu empfehlen.

Bezüglich der Nutzbarkeit im Realfall lässt sich festhalten, dass das Gesamtbild eher zweigeteilt war. Der komplette Ersatz eines Fachstabs in Präsenz durch eine alternative digitale Zusammenkunft wurde in den Auswertungen am kritischsten evaluiert. Jedoch ist unabhängig vom Format eine wichtige Schlussfolgerung, dass die Zusammenarbeit bei Routinen und beim Wissenstransfer innerhalb der beteiligten Organisationen und auch zwischen den Akteuren interorganisationell zu intensivieren ist. Die Zweifel an einer ausreichenden Realitätsabbildung bedeuten allerdings nicht, dass das digitale Format nicht die Präsenzübungen insbesondere dann ergänzen kann, wenn die gegebenen Umstände es erfordern. Es empfiehlt sich in jedem Fall, auf das entwickelte Konzept aufzubauen und weitere Verbesserungen regelmäßig einzupflegen.

Nichtsdestotrotz muss auf die limitierte Studiengröße hingewiesen werden, da die Gruppe der befragten Teilnehmenden im ARMIHN-Projekt mit einer Gesamtgröße von *n* = 20 insgesamt überschaubar war. Es ist daher kritisch zu hinterfragen, inwieweit unsere Erkenntnisse auch für größere Kollektive und Nutzergruppen Gültigkeit besitzen. An dieser Stelle soll jedoch ebenfalls darauf verwiesen werden, dass insbesondere bei den durchgeführten Übungsformaten nicht wesentlich mehr Teilnehmende indiziert gewesen wären. Eine viel größere Besetzung, bspw. der Fachstäbe, hätte zu einer weiteren Übungskünstlichkeit geführt. Es war jedoch ein wesentliches Ziel, keinen Bias unnötig zu verursachen. Die Auswahl der Teilnehmenden war eng an die resultierenden Anforderungen aus der Systemanalyse und den damit einhergehenden Stakeholder-Gesprächen am Projektanfang geknüpft worden. Auch die durchgeführten Experteninterviews haben gezeigt, dass innerhalb des Projekts eine erhebliche Fachexpertise angefragt und folglich genutzt werden konnte. Es ist daher zu unterstreichen, dass das gesammelte Feedback schon allein aufgrund der Ausgangssituation als besonders wertvoll zu betrachten ist. Auch wenn sich hierbei nicht eindeutig sagen lässt, ob ein tatsächlich vorhandener Effekt auch statistisch signifikant nachzuweisen ist, so genügen die gesammelten Informationen mit Sicherheit dazu, einen Überblick zu gewinnen. Auch ist eine Tendenz darstellbar bei der Klärung der Fragestellungen bezüglich Annahme des Systems, der Geeignetheit als Alternative, dem subjektiven Lerneffekt und der Geeignetheit der Online-Plattform für die Stabsarbeit im Realfall. Zudem ließ sich bezüglich der Konsistenz der Daten zeigen, dass die Kategorisierung und Bewertung schlüssig zu sein schien, da die Gruppierung ähnlich ausgerichteter (Kontroll‑)Fragen zwischen ausgewählten Einzelitems korrelierte, teilweise sogar mit statistischer Signifikanz.

**Figure 4:**
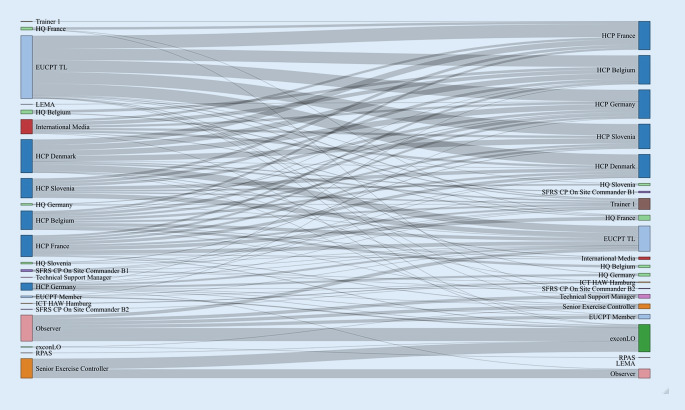

Die Online-Plattform hat sich zudem im Rahmen der Katastrophenhilfe bewährt. Die HAW Hamburg hat die Online-Plattform in leicht abgewandelter Form für das Projekt Digital ModTTX verwendet, welches Bestandteil des durch die EU geförderten Projekts Modules Table Top Exercises (N°ECHO/SER/2018/785702) war. Die COVID-19-Pandemie schränkte die Ausführung auch dieser Übungen ein, in denen unterschiedliche Szenarien durch sonst mehrfach pro Jahr stattfindende, nun fehlende internationale Reisen nicht wie gewohnt durchgeführt werden konnten. Daher wurden ein imaginäres Hochwasserszenario entwickelt und Videokonferenzräume etabliert. Dokumenten- und Informationsaustausch erfolgte wie im ARMIHN-Projekt über die digitale Übungsplattform. So konnte auch hier trotz Kontaktreduktion ein Realgeschehen simuliert werden. Eine Erweiterung war die grafische Darstellung und Analyse der E‑Mail-Kommunikations-Verläufe (Abb. 4). Diese Aufarbeitung und zukünftige Weiterentwicklungen der genutzten digitalen Plattform sollten auch für kommende Übungen noch tiefer greifende Einblicke in die Kommunikation einzelner Übungsteilnehmender bieten. Diese könnten weitere Anhaltspunkte für die Nutzerfreundlichkeit oder den Übungserfolg bieten.

Die dargestellten Ergebnisse bekräftigen hierbei die Annahme, dass sich das entwickelte System gut umsetzen lässt und als (Trainings‑)Alternative dienen kann. Als Motivation zur weiteren Digitalisierung auch in der zivilen Sicherheitsforschung sollte die Erkenntnis dienen, dass umso besser die digitale Umsetzung empfunden wurde, desto mehr profitierten die Teilnehmenden hinsichtlich des subjektiven Zugewinns ihrer Fähigkeiten. Auch wenn es sicherlich zukünftig anzustreben ist, das ganze System über einen längeren Zeitraum mit einer größeren Anzahl von Teilnehmenden weiter zu testen und validere Daten dabei zu gewinnen, so bestätigen die bereits durchgeführten Untersuchungen die hohe Endnutzerakzeptanz. Ein weiterer Vorteil ist die gute Übertragbarkeit der Plattform, da diese keiner besonderen technischen Voraussetzungen bedarf und durch die bewusste Auswahl gängiger Programme für andere Zwecke und Settings schnell adaptierbar ist. So ist eine Anpassung mit wenig IT-Aufwand und geringen Kosten im Rahmen einer Cloudlösung auch auf andere Projekte und Stabsübungen einfach umzusetzen. Zukünftig sollten weitere Untersuchungen noch relevante Daten zum Kosten-Nutzen-Verhältnis im Vergleich zur Präsenzveranstaltung bieten.

Kurz- und mittelfristig sind eine noch tiefer gehende Vernetzung und verbesserte Kommunikationsfähigkeit zwischen allen involvierten Akteuren wünschenswert. Langfristig ist durch regelmäßige Übungen insgesamt eine Erhöhung der Resilienz bezüglich des MANE im maritimen Setting zu erwarten. Zugleich sind mithilfe der digitalen Formate und Werkzeuge ebenfalls eine umfassende, zeit- und praxisnahe Skalierbarkeit sowie eine gezielte und detaillierte Auswertung der Übung möglich.

## Schlussfolgerung bzw. Fazit für die Praxis

Das digitale Format zeigte sich als geeignete Alternative bei der Umsetzung von Simulationsübungen unter Pandemiebedingungen, insbesondere auch organisatorisch und finanziell. Vorteilhaft ist die Reduktion von Personal- und Folgekosten, da der hohe Personaleinsatz im Gegensatz zu Präsenzübungen überschaubarer bleibt, hauptsächlich die Anfangsinvestitionen als Fixkosten zu beachten sind und wenig Folgekosten resultieren. Übungen lassen sich folglich einfacher terminkonform durchführen. Auch aus infektiologischer Sicht ist der digitale Ansatz besonders angesichts der Vermeidung von Infektionsketten sinnvoll. Darüber hinaus verwendet die HAW Hamburg seit 2020 im Studiengang Gefahrenabwehr das digitale Format über die Plattform in der Lehre zur Simulation von Stabsrahmenübungen, was ein weiteres Anwendungsgebiet darstellt.

Bezüglich des längerfristigen Potenzials zur Steigerung der Resilienz durch digitale Simulation sollte eine Weiterentwicklung und Evaluation dieser Alternative notwendigerweise erfolgen. Somit könnte sichergestellt werden, dass sich Einsatzkräfte und involvierte Personen mit Übungen auf Ereignisse wie einen MANE vorbereiten und die jeweiligen Gefahrenabwehrsysteme testen können, um die hoheitliche Aufgabe der Gefahrenabwehr auch in Zukunft möglichst trainiert und routiniert sicherstellen zu können.

## Supplementary Information




