# Supplementary figures and images for: Virtual staff teamwork during the pandemic – development of digital training formats for community emergency response during the coronavirus pandemic: Experience using the example of the ARMIHN project
Source: Notf Rett Med. 2023 Jun 15:1–10. [Article in German] Online ahead of print. doi: 10.1007/s10049-023-01164-7 (PMC10267551; doi:10.1007/s10049-023-01164-7)

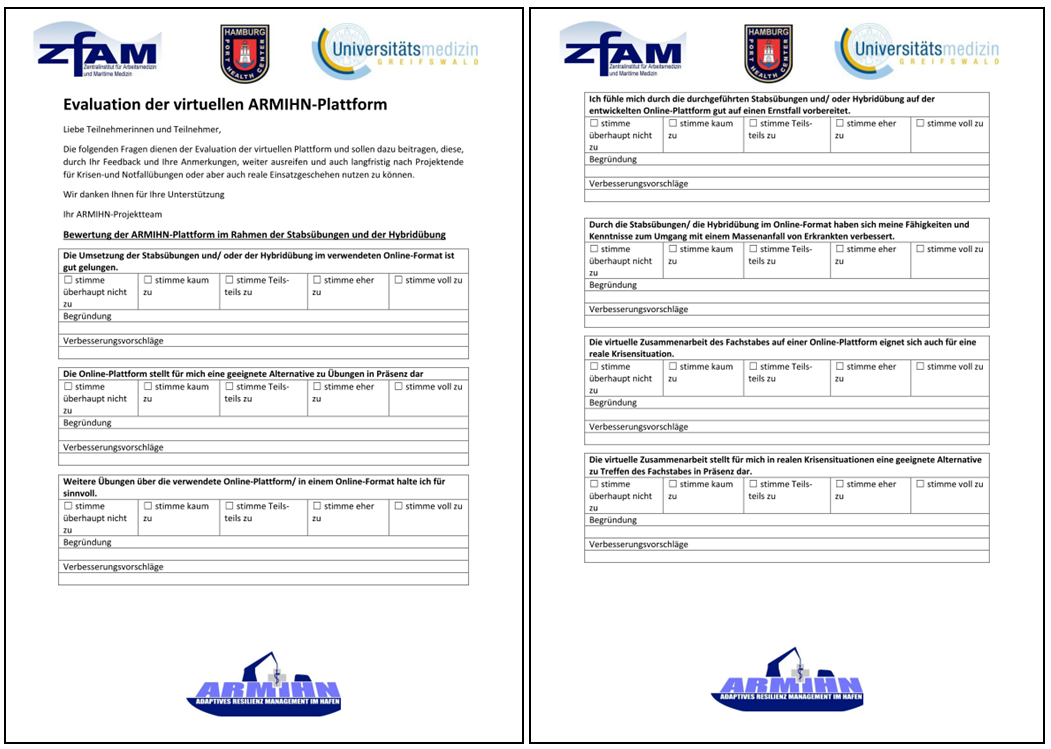

Supplement: Supplementary file 1 [file 10049_2023_1164_MOESM1_ESM.png]
